# Trichothecenes in Cereal Grains

**DOI:** 10.3390/ijms10010147

**Published:** 2009-01-06

**Authors:** Nora A. Foroud, François Eudes

**Affiliations:** 1 Lethbridge Research Centre, Agriculture and Agri-Food Canada, 5403 1^st^ Avenue South, Lethbridge, AB, Canada T1J 4B1. E-Mail: nora.foroud@agr.gc.ca; 2 Michael Smith Laboratories, The University of British Columbia, #301 - 2185 East Mall, Vancouver, B.C., Canada V6T 1Z4

**Keywords:** Trichothecene, fusarium head blight, cereal grain

## Abstract

Trichothecenes are sesquiterpenoid mycotoxins associated with fusarium head blight (FHB) of cereals, with worldwide economic and health impacts. While various management strategies have been proposed to reduce the mycotoxin risk, breeding towards FHB-resistance appears to be the most effective means to manage the disease, and reduce trichothecene contamination of cereal-based food products. This review provides a brief summary of the trichothecene synthesis in *Fusarium* species, their toxicity in plants and humans, followed by the current methods of screening and breeding for resistance to FHB and trichothecene accumulation.

## 1. Introduction

Fusarium head blight (FHB) is a destructive disease of cereal grain crops, with worldwide economic impact. The disease is caused by a series of trichothecene-producing *Fusarium* species, of which *F. graminearum* (teleomorph: *Gibberella zeae*) and *F. culmorum* are the most economically relevant [1,2]. Trichothecenes are sesquiterpenoid mycotoxins that have been implicated in disease aggressiveness [4-6] and are found to accumulate in kernels of infected spikelets, rendering the grain unsuitable for human or animal consumption. Ingestion of contaminated grain can cause intestinal irritation in mammals, and can lead to feed refusal in livestock [7]. A few outbreaks of alimentary toxic aleukia (ATA), a potentially fatal condition caused by trichothecene ingestion, have been reported in human societies as far back as the 18^th^ century (see Section 2.2). The main source of mycotoxins in the food chain is *Fusarium*-contaminated grain, usually from FHB-outbreaks, although some species may proliferate on grain during storage. The first documented FHB-outbreak occurred in England in 1884, where the disease was named “wheat scab” [8]. Outbreaks have since been reported in the Americas [9–11], Asia [12, 13], Australia [14], Europe [1, 15], and South Africa [16, 17]. The most notorious epidemic in North America spanned the 1990s, where in the United States alone, estimated economic losses approached 3 billion USD [18]. In South Africa, a double cropping system (with maize as a summer crop and wheat as a winter crop) in combination with conservation tillage has led to a growing FHB problem, especially in regions where irrigation is required [16]. Rice-wheat rotations are routine in many Asian countries. Both rice and wheat are host-crops, and while FHB is more endemic in the latter, the former still serves as a host for innoculum buildup [18]. FHB of barley and wheat is a pervasive problem in China. Between 1951 and 1990, wheat farmers were burdened with seven severe epidemics (exceeding 40% yield losses) and 14 moderate epidemics (10–20% yield losses) [12]. The subsequent introduction of moderately resistant cultivars has coincided with a reduced frequency of severe epidemics [20].

FHB-management strategies are essential for reducing the economic damages and potential health hazards associated with this disease. Strategies have been developed to target each of the three components (innoculum source, susceptible host, and favorable environmental conditions) which are necessary for an outbreak. Innoculum source is usually present in the form of ascospores in the soil. Studies have shown that crop-rotation, tillage, chemical or biological control, and the use of FHB-resistant cultivars can all contribute to reduce the amount of innoculum harbored in the soil by reducing the amount of *Fusarium/Gibberella*-contaminated crop and/or crop debris [21–27]. The second essential component for an outbreak is a suitable host for the spores to germinate and establish disease. In order to reduce the impact of this disease, we need to convince farmers to grow resistant cultivars. The selection of registered cultivars with decent FHB-resistance is limited. Moreover, farmers preferentially select cultivars with good agronomics, which often have poor resistance. Finally, the third component necessary for disease establishment: favorable environmental conditions. FHB thrives in wet, humid conditions with an optimum temperature of 25°C during anthesis and grain filling stages of crop development [1]. While the weather cannot be controlled, disease forecasting models can be used to devise an effective spraying schedule for chemical control, and for more organized post-harvest management of potentially diseased kernels [10, 19, 28–29]. Application of more than one management strategy [30] has been shown to be most effective in reducing FHB severity and trichothecene accumulation in grains. However, if we had to focus our efforts on only one disease management strategy, the development of highly resistant cultivars, with good agronomic qualities, would have the largest impact. The use of resistant cultivars can manage each of the three components essential for disease. First, it can reduce the amount of inoculum buildup in host-crop debris (and subsequently in the soil). Second, short of only cultivating non-host crops, we can reduce the suitability of host-crops by using highly-resistant cultivars. Finally, while a strong genotype-environment interaction has been observed in FHB disease outcomes [31], studies have shown that the effect of the environment can be indirectly managed by using highly-resistant genotypes. In contrast, while genotypes with moderate or intermediate resistance can reduce the impact of FHB, this resistance is not stable under high disease pressure.

In a two-year FHB field trial of winter wheat, rye and triticale genotypes, grown in three regions of South-West Germany, Miedaner *et al*. (2001) observed strong interactions between genotype and disease outcomes/trichothecene accumulation [31]. They also observed a significant impact of the environment on these interactions, but the effect of the environment was reduced in more resistant genotypes. We observed a similar phenomenon between wheat-genotypes and their interaction with different *F. graminearum* trichothecene-chemotypes (chemotype meaning the major trichothecene produced by a given strain) [32]. Trichothecene-chemotype played a role in disease spread and in the development of Fusarium damaged kernels (FDK) in intermediate/moderate sources of resistance, but the effect of chemotype was not observed in susceptible and resistant genotypes. High levels of resistance or susceptibility were stable across all chemotypes tested [32]. We also found that while the 3-*O*-acetyl 4-deoxynivalenol (3ADON) chemotypes led to the highest disease symptoms in intermediate/moderate resistant sources, FDK in plants infected with 3ADON-producers was nearly as low as the least aggressive chemotype, nivalenol (NIV). But, again, in highly resistant sources, the aggressiveness of the isolate and the FDK did not impact disease outcomes.

These studies emphasize the importance of developing highly resistant cultivars. The level of resistance “is more important in governing [4-deoxynivalenol (DON)] accumulation in a given cultivar than is the aggressiveness of an isolate [33]”. In this review, we will begin with an overview of trichothecenes to emphasize their impact on FHB-disease outcomes and on human health. This will be followed by a section on FHB-resistance, sources and current breeding methods. Finally, we will conclude with our recommendations of the direction breeding programs should take in order to have the highest impact on reducing FHB of grain crops and trichothecene accumulation in the food chain.

## 2. Trichothecenes

### 2.1. Trichothecene biosynthesis and structure

**Figure 1.**
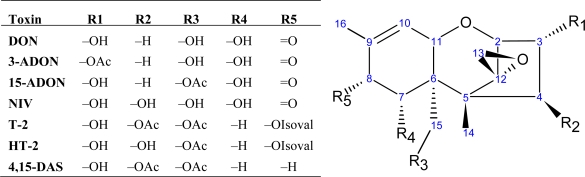
Type A and B trichothecene structures. Examples of type A trichothecenes include T-2 toxin (T-2), HT-2 toxin (HT-2), 4,15-diacetoxyscirpenol (4,15-DAS). Examples of type B trichothecenes include nivalenol (NIV), 4-deoxynivalenol (DON), 3-*O*-acetyl DON (3-ADON), and 15-*O*-acetyl DON (15-ADON). OAc = acetyl function; OIsoval = isovalerate function.

**Scheme 1.**
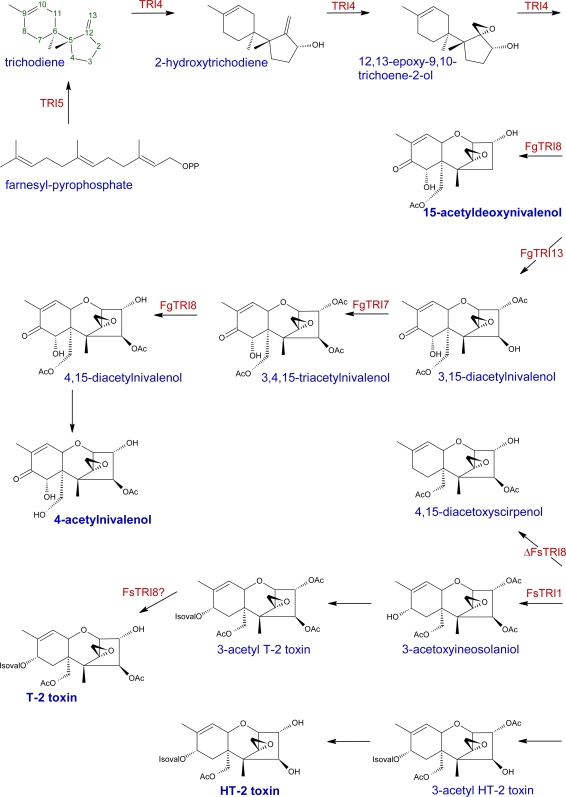

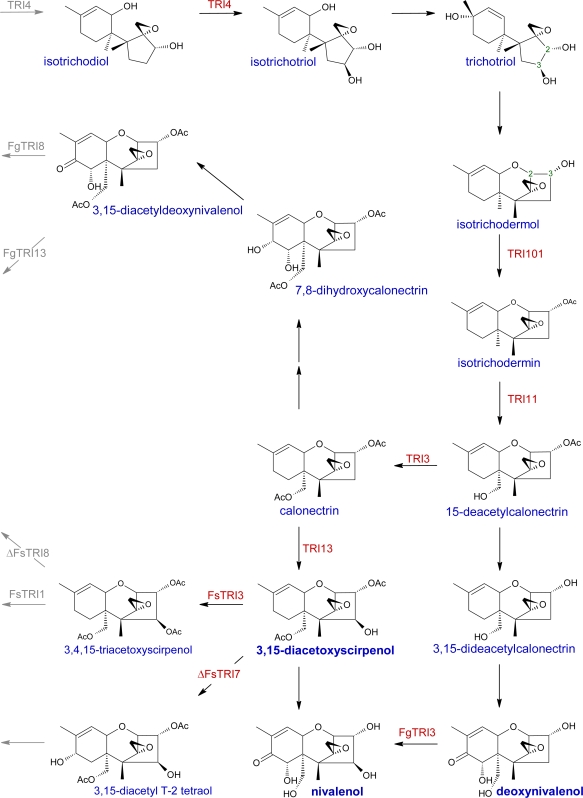
Trichothecene biosynthesis pathways. Steps in the pathway are catalyzed by *Tri*-gene products (see Table 1 for more details), and have been identified in either *F. graminearum* (Fg), *F. sporotrichioides* (Fs), or both. OAc = acetyl function; IsovalO = isovalerate function. Diagram is modified from [33, 50, 169, and 172].

Trichothecenes are toxic sesquiterpenoid compounds composed of a central core of fused cyclohexene/tetrahydropyran rings. In addition, a cyclopentyl moiety is also fused to the tetrahydropyran ring through C-2 and C-5. Furthermore, C-12 comprises part of an epoxide functionality (Figure 1), which has been deemed crucial for toxicity [34]. There are five positions at which functionality varies, most commonly featuring hydroxyl or acetyl groups. Four types of trichothecenes have been identified from trichothecene-producing fungi: types A, B, C and D. The major type A trichothecenes in *Fusarium* species include T-2 toxin (T-2) and HT-2 toxin (HT-2), both of which posses an isovalerate function at C-8 [35]. *F. sporotrichiodies* and *F. poae* are some of the major type A trichothecene producers [36]. Type A trichothecenes are highly toxic; T-2 has been reported to be roughly ten times more toxic in mammals than DON [37]. DON is the most prevalent toxin associated with FHB, and belongs to the more phytotoxic [3] type B trichothecenes which feature a ketone at C-8 [35]. *F. culmorum* and *F. graminearum* produce mainly DON, NIV and their derivatives [36]. Type C and D trichothecenes, respectively characterized by a second epoxide (C-7,8 or C-9,10) or an ester-linked macrocycle (C-4,16), are not associated with FHB [38]. Other mycotoxins, such as zearelenone (ZON), fumonisins, moniliformin and butenolide are also produced by *Fusarium* species [35, 39-40]. The trichothecene biosynthesis pathway is summarized in Scheme 1; genes associated with trichothecene production are presented in Table 1. The initial substrate, farnesyl pyrophosphate, is cyclized into a non-toxic trichothecene product, trichodiene. This step is catalyzed by trichodiene synthase, TRI5 [6, 34, 41]. A multifunctional cytochrome P450 monooxygenase, TRI4, catalyzes the next four steps in the pathway: C-2 hydroxylation [42–43], followed by 12,13 epoxidation and two more hydroxylation reactions [43]. The final product of TRI4 activity, isotrichotriol, undergoes two non-enzymatic isomerization steps [44], including a cyclization whereby C–O bond formation occurs between the C-2 oxygen and C-11. The product, isotrichodermol, forms the skeleton trichothecene structure, and is acetylated by TRI101 at C-3 [45], and hydroxylated by TRI11 at C-15 to produce 15-deacetylcalonectrin [46]. 15-deacetylcalonectrin can act as a substrate for DON production, by hydroxylation of C-3 and C-7 and the addition of a ketone group at C-8. Alternatively, it can be acetylated by the activity of TRI3 at C-15 to produce calonectrin [47].

Calonectrin serves as a substrate for the biosynthesis of acetylated DON and NIV products, including 3,15-diacetyldeoxynivalenol (3,15-ADON), 15-acetyldeoxynivalenol (15-ADON), and 4-acetylnivalenol (4-ANIV). Functional expression of *F. graminearum Tri*7 and *Tri13* genes is necessary for NIV-chemotypes of this species—the absence of these genes confers DON-chemotypes [48–49]. NIV can be synthesized directly from DON, or by conversion of calonectrin to 3,15-diacetoxyscirpenol (-DAS) and then to NIV by ketone addition at C-8. 3,15-DAS is also the major substrate type A trichothecenes. Functional expression of *F. sporotrichioides Tri7* and *Tri8* produces T-2 toxin chemotypes in this species [50]. C-4 acetylation of 3,15-DAS (catalyzed by TRI7) produces 3,4,15-triacetoxyscirpenol, which can be converted to 4,15-DAS by TRI8, or serves as substrate for T-2 toxin synthesis, initiated by TRI1. HT-2 toxin accumulates in the absence of TRI7, via hydroxylation and isovalerate addition to C-8 of 3,15-DAS.

### 2.2. Trichothecene Toxicity: Food Safety and Quality

Trichothecene exposure can lead to growth retardation in eukaryotes, causing reproductive dysfunction in mammals and inhibition of seedling growth/regeneration in plants (reviewed in [51]). The toxicity of trichothecenes is attributed to their ability to inhibit peptidyl transferase activity of 60S ribosomes [52]. Additional impacts of trichothecene toxicity (reviewed in [51, 53, 54] include disruption of nucleic acid synthesis [55, 56], mitochondrial function [55, 57], membrane integrity [58, 59], and cell division. Trichothecenes have been shown to induce apoptosis in animal cells [60–62], and may induce programmed cell death in plants [51].

It is pertinent that regulations be put into place to control the allowable quantities of trichothecenes entering the food chain. Ingestion of contaminated grain can cause alimentary toxic aleukia (ATA); a condition characterized by an initial stage of intestinal irritation causing emesis and diarrhea, followed by aleukia and anemia, and which may ultimately lead to death. The first recorded *Fusarium*-related ATA outbreak occurred in Siberia in 1913, but the human impacts of ATA may go as far back as the 1730s, when symptoms of a reported disease epidemic in New Hampshire are reminiscent of ATA (reviewed in [8]). The most devastating outbreak occurred in Russia between 1942 and 1948, where at least 100,000 people died. In this case, over-wintered grain had become contaminated with T-2 producing *F. sporotrichioides* or *F. poae*, during mild winters [63, 64]. It was not until 1950 that the connection between *Fusarium* toxins and ATA was established.

The most abundant source of trichothecene contamination in cereal grains today is due to FHB, which is primarily caused by type-B trichothecene-producers. Trichothecene accumulation occurs when spikes are infected during or post-anthesis [65], although significant yield losses are more relevant in early infections (anthesis to early stages of kernel development) [66–67]. Trichothecene-contaminated grain is readily distinguished owing to its shriveled and discolored appearance. Several studies have shown that percentage of Fusarium damaged kernels (FDK) serves as a reliable estimate of DON content [31, 33]. This allows for rapid visual screening of Fusarium damaged kernels (FDK) in order to prevent contaminated grain entering the food chain in unsafe quantities—although FDK screening for toxin contamination should be used with caution since reduced physical damage is observed if infection occurs past the soft dough stages of kernel development [65, 68]. FDK can be used as a preliminary screen when sorting and grading grain, but quantitative trichothecene testing is advisable before the grain enters the food chain. In Canada, maximum FDK limits are in place for different wheat classes and varieties, and are enforced by the Canadian Grain Commission at licensed grain elevators. DON-testing is carried out on end-products by the Canadian Food Inspection Agency and Health Canada to ensure the maximum allowable in quantity of this trichothecene in food-stuffs is not exceeded. Currently, the maximum limit of DON is set at 2 ppm for Canadian soft wheat (1 ppm for use in baby food), and the establishment of maximum limits of DON in hard wheat is currently being evaluated (Tom Nowicki and Randy Clear, personal communications). The established DON limits are also being reviewed, and more rigid standards may be imposed to harmonize the standards with those of the European Union. DON tolerance in China, Hungary, Russia, Switzerland, and the United States is 1 ppm; and in Austria, Germany, and the Netherlands is 0.5 ppm [69]. Standards are also put into place for grain that is used in animal feed, as the trichothecenes cause similar ailments in farm animals as in humans [70].

### 2.3. Trichothecenes as Aggressiveness Factors in Fusarium Head Blight

Different levels of aggressiveness and pathogenicity have been observed in different isolates of a given *Fusarium* species. These differences can be attributed only in part to fitness, suggesting that aggressiveness factors may contribute to disease outcomes. Toxins have long been implicated as aggressiveness factors in pathogen systems. Examples include host selective toxins (HSTs), which are produced by the pathogen, are only toxic towards the target host, and are necessary for causing disease [reviewed in 71]. These include AK-toxin of *Alternaria alternata*, victorin of *Cochliobolus victoriae*, and T-toxin of *C. heterostrophus*, the causal agents in black spot of Japanese pear [72], Victoria blight of oats [73], and corn leaf blight [74], respectively. Other examples of toxins involved in pathogen aggressiveness or pathogenicity in plants include botryane of grey mould causing *Botrytis cinerea* [75] and zinniol of *Alternaria* species [76]. Over the past few decades, evidence of trichothecenes as non-HSTs involved in aggressiveness of *Fusarium*-related diseases has been accumulating. Observations by Beremand *et al*. (1991) suggested a link between trichothecene production, fertility and pathogenicity of *F. sambucinum* isolates [77]. Correlations between fungal biomass (measured by ergosterol quantification) and DON accumulation in cereal grains have been observed [31, 78–79]. A link between DON accumulation and disease outcomes has also been observed [68, 80]. The trichothecene-chemotype of isolates [32] and the cumulative impact of multiple trichothecenes produced either by a single isolate [81] or by a composite of isolates [82] may increase disease severity. In addition, some FHB-resistant sources have been shown to have the ability to detoxify DON, primarily by glycosylation [reviewed in 83]. Together, these data suggest that the ability of *Fusarium* species to cause disease is linked to trichothecene accumulation in the host, and that reduced aggressiveness may be observed by either reduced toxin production by the pathogen, or removal/degradation of the toxin by the host.

In 1995, Proctor *et al*. developed a trichothecene non-producing strain of *F. graminearum* (Tri5-; wild-type Tri5+), prepared by gene disruption of trichodiene synthase (*Tri5*) [6]. A series of studies have been conducted using these strains, in order to clarify the role of trichothecenes in FHB-aggressiveness, and results are generally consistent with a role for trichothecenes in disease spread in *Triticeae* [3, 5–6, 84–85) and in maize [4]. Trichothecenes are not necessary for initial infection [84], or infection of the wheat fruit coat, but they are required for entry into the rachis and subsequently for disease spread [86]. In the absence of trichothecene-production, *F. graminearum* is shown to be contained in point-inoculated spikelets by cell wall thickening at the rachis node [86].

## 3. Fusarium Head Blight Resistance

### 3.1. Mechanisms of Resistance

FHB resistance is dominant and quantitative. A gene-for-gene resistance interaction has not been identified in FHB-resistance, and immunity to the disease has not been observed. Stability of resistance is dependent on environmental-factors at the time of infection and/or aggressiveness-factors associated with the invading *Fusarium* strain—although resistance has been shown to be stable in genotypes with very high levels of resistance [31–32, 87]. Several different forms of resistance have been identified (Table 2). These mechanisms of resistance can interact with each other to improve the overall resistance.

Due to the physiological differences between maize and other cereals, resistance in maize is described separately. Two major forms of resistance in maize include: (1) silk-resistance, where the fungus cannot penetrate the silk channel to infect the kernels [88]; and (2) kernel-resistance, where the fungus cannot penetrate the rachis, or ‘cob’, and hence does not spread from kernel to kernel [89]. These two forms of resistance in maize are somewhat similar to resistance to initial infection (type I) and disease spread (type II), respectively, in other cereal grains. Type I and type II resistances, first described by Schroeder and Christensen, are the best documented forms of resistance, since they are the most readily ascertained [90]. Type I resistance can be measured as the percentage of spikelets exhibiting symptoms upon exposure to the pathogen. Plants are typically sprayed during anthesis with macroconidial (or ascosporic) suspensions and high-humidity is maintained (by bagging infected heads or by mist-irrigation) for a few days after inoculation. Alternatively, the grain spawn method, where infected wheat or corn is dispersed in the field, can be used to better mimic natural conditions of infection [91–92]. Resistance is measured 7 to 21 days after anthesis, typically reported as a ‘disease index’, where ‘incidence’ (percentage of diseased spikes) is multiplied by ‘severity’ (percentage of infected spikelets on diseased spikes). Acquiring accurate assessments of type I resistance is impeded by several factors: (a) the amount of inoculum that actually reaches the spikelets is immeasurable, resulting in variability in the exposure of different spikes or plants within and between experiments; (b) environmental conditions are difficult to control, especially in field experiments; and (c) ‘disease index’ is not a measure of type I resistance alone, but rather a combination of resistance to initial infection, disease spread and tolerance [92–93]. Some researchers equate ‘incidence’ with type I resistance and ‘severity’ with type II resistance, while others equate ‘disease index’ as an estimate of both type I and II resistances [92]. These inconsistencies in evaluation standards stress the need to review our definition of type I resistance, as is proposed by Mesterházy [94].

Evaluation of type II resistance is a little more straightforward [92]. A quantifiable amount of inoculum is injected into individual spikelets at anthesis, and high-humidity maintained for several days. Resistance is measured as the number of infected spikelets below the inoculation point; note that the disease typically spreads down the spike through the rachis. Delayed hyphal colonization of the vascular bundles in the rachis is observed in type II resistant genotypes [95]. Recent work presented by Ilgen *et al*. shows that trichodiene synthase expression is induced when the growing hyphae comes in contact with the ovaries [96]. It is likely that metabolite(s) present in the ovaries induces expression of trichothecene synthesis. We already know that trichothecenes are necessary for disease spread [3, 5–6, 84], but they do not appear to play a role in establishing initial infection by spray or point inoculation [84]. The data presented by Ilgen *et al*. effectively demonstrates that trichothecene synthesis does not begin until the fungus has successfully invaded the spikelet [96]. In other words, trichothecenes, which are necessary for disease spread, do not accumulate until after initial infection has been established.

If trichothecenes are not necessary for establishing initial infection, and since evaluation methods for resistance to initial infection is confounded by resistance to disease spread, then perhaps a more accurate estimate of resistance to “initial infection” would be by spray inoculation with Tri5-, or even by using grain spawn infected with Tri5-. This would eliminate the interference of type II and III resistances in our evaluation of resistance to initial infection. On the other hand, in addition to the remaining problems or difficulties in quantifying spore exposure and controlling environmental conditions, using Tri5- to test for type I resistance would be accompanied with its own set of constraints. The Tri5- strain is a valuable tool for addressing fundamental research questions, but is not as functional in applied research. Breeders will still need to address the other components of resistance, and while the evaluation of type I resistance is confounded by other forms of resistance, breeders and farmers may be interested in these other forms of resistance—they want cultivars that are resistant to spray inoculation and to all that entails. They want crops that are resistant to the disease, not to components of the disease. While good type I resistance may be the most effective means to prevent the disease from occurring, these genotypes are not immune to the disease, and the additional components of resistance will therefore play an important role in crop protection.

The three remaining forms of resistance (type III, IV, and V) cannot be quantified directly. This is in part, because the nature of these forms of resistance is often intermingled with each other and/or with type I and II resistances. Type III resistance can be assessed by FDK evaluation. Type IV resistance is defined as tolerance to FHB, meaning that yield and quality is maintained despite disease presence. Type IV resistance may also be defined as tolerance to DON, in which case it can be evaluated by comparing FDK values to DON content; if FDK is low but DON content is high, tolerance to DON and FHB would be observed. Type V resistance (resistance to toxin accumulation) can be estimated by DON quantification of FHB-infected plants or by an *in vitro* tissue assay [97–98]. Type V resistance can be subdivided into two classes (types V-1 and V-2) as recently defined by Boutigny *et al*. [83]. In type V-1 resistance, plants are able to chemically modify trichothecenes, resulting in toxin degradation or detoxification. Type V-2 resistance refers to genotypes that have the ability to inhibit trichothecene biosynthesis in the invading fungus.

### 3.2. Sources of Resistance

The development of FHB-resistant cultivars has proven to be a difficult task. While cereal breeders worldwide have invested a considerable effort in the development of FHB-resistant germplasm [9, 12, 99–102], relatively few resistant cultivars have been generated by conventional breeding methods. Moreover, most of the work has focused on wheat and barley breeding. This is a direct result of the FHB-impact on wheat and barley (especially wheat) in comparison to other cereals. Wheat is one of the most heavily FHB-affected crops and accumulates the largest economic damage. Rye is generally more resistant than wheat and barley [5, 100]. Oats are also more resistant than wheat and barley [5], but DON accumulation in oats is more severe than in wheat [103]. T-2 and HT-2 toxin accumulation has been observed in oats in Norwegian countries, due to infection with head blight causing *F. langsethiae* [104]. Higher apparent FHB-resistance in oat is, in part, due to difficulties in screening for resistance. Disease symptoms of *Fusarium*-infected standing oat are not as readily discernable as in wheat or barley where symptoms are clearly visible [102].

Barley has an inherent type II resistance [5], but unconventional disease spread can be observed externally from spikelet to spikelet without penetration of the rachis [5]. Six-row barley, which is more susceptible than two-row barley and is preferred for malting, is nearly as susceptible as wheat [101]. Chevron, the major source of resistance in six-row barley, is not well-liked in the brewing and malting industries due to elevated protein content [105]. QTL for FHB-resistance have been found on all seven barley chromosomes [106–108]. Resistance in two-row barley is attributed to a QTL that is associated with the Vrs1 locus, which controls spike type. It is not clear whether resistance is linked to Vrs1, of if there is a pleiotropic effect at play.

Durum wheat (*Triticum turgidum* subsp. *durum*) is one of eight subspecies of tetraploid (AABB) wheat [109], and is far more FHB-susceptible than hexaploid (AABBDD) bread wheat [9]. Breeders have screened for resistant sources in various *T. turgidum* subspecies. Wild emmer wheat (*T. turgidum* subsp. *dicoccoides*) has been the major focus for alternative tetraploid wheat resistance; however, poor agronomic traits have prevented its use in breeding programs. Recently, Oliver *et al*. took on the task of systematically screening seven *T. turgidum* subspecies, and have identified some with promise as type II resistant sources [109]. Four QTL have been identified for FHB-resistance in tetraploid wheat: 3AS and 7AS from different accessions of *T. turgidum* subsp. *dicoccoides*, and 2BS and 6BS from durum wheat [110].

Over 100 QTL have been reported from FHB-resistant wheat sources—22 of which have been found in multiple mapping populations, and are nicely summarized in a recent review by Bürstmayr *et al*. [110]. The best characterized and most widely used source of resistance in hexaploid wheat is the Chinese cultivar, ‘Sumai3’ [110–111]. ‘Sumai3’-derived resistance is attributed to the Fhb1 locus on chromosome 3BS, the major QTL conferring type II resistance. Positional cloning of Fhb1 is underway, and gene identification may become available within the next year or so [112]. Additional QTL from ‘Sumai3’ include Fhb2 (6BS) and Qfhs.ifa-5A (5A), the latter being associated with type I resistance and found in different germplasm from around the world [110]. Another popular resistance source comes from the Brazilian cultivar, ‘Frontana’, with moderate resistance, and QTL mapped to 3A, 5A, 2B, 3AL, and 7AS [110, 113–115]. An interesting set of QTL for FHB-resistance may be associated with Rht plant height regulators. Some studies have shown that plant height is correlated with FHB-resistance [115–117], although rare exceptions may be found (A. Comeau, unpublished data). Rht-D1 co-localizes with FHB-resistant QTL on 4DS, found in the European winter wheat cultivar, Arina [118]. Rht-B1 is on the same chromosome as an FHB-resistant QTL on 4B, and Rht8 is close to a QTL on 2D. Rht-B1 and Rht-D1 are derepressors of gibberellin-signaling, and Rht8 is gibberellin-responsive. The so-called semi-dwarfing alleles, Rht-B1b and Rht-D1b, confer gibberellin-insensitivity [119]. Rht-D1b, but not Rht-B1b, also confers FHB-susceptibility [118, 120]. It has not yet been determined whether or not there is a pleiotropic effect at play in this interaction [118].

Research in fusarium ear blight (FEB; FHB of maize)-resistance in maize is less extensive than in wheat and barley, and few resistant cultivars have been generated. Canadian inbred lines with resistance include CO272 [moderate silk resistance; 89], CO325 [moderate kernel resistance; 89] and CO441 [with both silk and kernel resistance; 121]. To our knowledge, only two studies have been published in the identification of QTL for FEB-resistant maize [122–123]. Ali *et al*. identified 11 QTL for silk-resistance and 18 QTL for kernel-resistance, two of which were also associated with silk-resistance [122]. Microarray-based comparative genomic hybridization is currently underway to identify genes corresponding to those QTL [124]. It is essential that more research be put into FEB-resistance in maize, maize is a major staple food and production has increased in the United States due to the biofuel initiatives. As a result of increased maize production we should expect to see an increase in inoculum build-up in the soil, and maize is one of the worst culprits for harbouring ascospores. By improving maize resistance to FEB, we will not only protect maize crops, but indirectly protect other crops that would be affected by the aforementioned potential increase in inoculum build-up. This is, in fact, an issue of food security. The increase in maize production for non-food products is already taking away valuable farmland for food production, thus reducing the quantity of wheat and barley, among other crops. If the soil-borne inocula were to increase, this would threaten the already dwindling food supply of wheat and other grains. This issue also reiterates the importance of producing highly resistant cultivars of all the other susceptible cereals.

### 3.3. Breeding for Resistance

Some of the challenges breeders are confronted with include: (1) poor agronomic traits associated with highly-resistant germplasm, which are often derived from exotic sources, (2) the polygenic nature of resistance, and (3) variability in disease rating such as those described in section 3.1. Unfortunately, breeders have been limited in their choices of resistant sources, and some of these problems perpetuate themselves. For example, while gene-for-gene resistance is presented with its own set of difficulties [such as the development of an arms-race between host and pathogen; 125], the lack of vertical resistance in FHB-host interactions precludes the prospect of immunity. As a consequence, when breeders find stable, highly-resistant sources, they use this source of resistance—as they should. However, if there are only a handful of these resistant sources available, then they will be used over-and-over again, ultimately leading to the arms-race scenario observed in gene-for-gene resistance. Limiting breeding programs to one (or a few) resistant sources can initiate the selection of highly pathogenic strains with the ability to disarm or dilute the resistant strategy of cultivated lines. This limitation brings us back to the original problem: FHB-resistance is quantitative. Therefore, the use of only a limited number of resistant sources in the development of resistant germplasm is not terribly effective. In addition, most of these resistant sources have poor agronomic traits—and so you can see the cycle continuing. ‘Sumai3’ is the prime example of a stable resistant source that is used in breeding programs around the world. The stability and level of resistance is higher than in any other registered cultivar. Other popular resistant sources exist (ex. ‘Frontana’), but there are only a few of them. In addition, ‘Sumai3’ is blighted with several agronomic fallacies, including susceptibility to kernel shattering and reduced yields [126].

In the past few decades, several tactics have emerged in an effort to enhance, or as an alternative to, traditional breeding methods. Traditional breeding is a long and tedious process, requiring many generations of screening, which can be costly and time consuming. Breeders often screen thousands of lines, narrowing them down every year, for up to ten years, before one or two lines are ready for the application process to register a cultivar. Screening for FHB-severity alone is time consuming, but screening for trichothecene accumulation (which is a necessary step) is both time-consuming and costly. Several studies have shown that percent FDK serves as a reliable estimate of DON content [33, 127]. For this reason, and because it is rapid and inexpensive, FDK is frequently used to screen for resistance to DON accumulation in breeding programs. Since FHB-severity is highly correlated with seed quality and DON content (127), it is recommended that breeders screen only for FHB-severity in the early years, and then use DON quantification when the number of breeding lines has been narrowed down [31].

The use of QTL (such as those described in section 3.2) as molecular markers for resistance can facilitate breeding in the early stages of screening, and can effectively attenuate some of the error in the disease rating variability. Other methods have been proposed to decrease the occurrence of these errors in rating disease, in particular indoor spray inoculation which mimics natural infection [128]. The indoor spray inoculation method using a mist-irrigated greenhouse allows for a relatively rapid screening of advanced material, as well as screening of earlier F_2–4_ generations and doubled haploid genotypes. Indoor spray inoculation also offers the possibility of running phenotypic evaluations throughout the calendar year, which is not possible in a nursery, and is more likely to prevent competing organisms (e.g., *Bipolaris sorokiniana* (Sacc.) Shoemaker) from confounding the evaluations.

The idea of using trichothecenes as factors for early selection of resistant cereal lines, or reduced accumulation of mycotoxins, is very attractive. Large cell samples can be evaluated in Petri dishes, where environmental conditions are uniform. Bruins *et al*. suggested that single-cell microspores might be better suited for *in vitro* selection since a large proportion of the plant genome is expressed in both the sporophyte and the gametophyte [129]. Fadel and Wenzel were the first to report a mixed culture filtrate of 99 *F. graminearum* isolates co-cultured in 10-day old anther cultures [130]. However, they did not report successful selection for resistance among regenerated plants. In a similar study, Eudes *et al*. also co-cultured crude trichothecenes in an anther culture assay to report effective selection for reduced mycotoxin accumulation when using the more defined selection agent [131]. This *in vitro* selection process was successful using source of resistance Frontana, and was genotype dependant.

### 3.2. Engineering for Resistance

Several reviews have been published on the advantages of genetic engineering toward improvement of crop resistance to various pathogens, including FHB [132–134]. Genetic engineering towards FHB-resistance can be effective in reducing mycotoxin accumulation in cereals. Transgenic crops can directly reduce trichothecene accumulation when the modifications include expression of genes or pathways within the host that inhibit trichothecene synthesis, detoxify trichothecenes, or remove them from the cell by efflux. Boutigny *et al.* recently published a review summarizing the known methods naturally expressed by plants to reduce trichothecene accumulation [83]. Examples of enzymes that may be involved in detoxification include epoxidases, acetyltransferases, and glycosyltransferases [83]. While there is no evidence of de-epoxidation *in planta* [83], the epoxide group has been shown to be essential for trichothecene toxicity [135]. Trichothecene 3-*O*-acetyltransferase activity of TRI101, which leads to DON-acetylation, has been shown to reduce toxicity of trichothecenes in *F. graminearum* [136]. It has also been demonstrated that 3-ADON is less cytotoxic than DON [137]. Transgenic expression of TRI101 in plants has shown improved FHB- and trichothecene-resistance [138–140]. Another *Fusarium* gene, *Tri12*, is a major facilitator superfamily (MFS) transporter responsible for trichothecene efflux from *F. sporotrichioides* [141], also has the potential to reduce trichothecene accumulation in plants.

UDP-glucosyltransferase has been shown to detoxify DON, by condensation of a glucose molecule on the hydroxyl group at C-3 [142], and glycosylated-DON derivatives have been observed in *Fusarium*-infected cereals [143–145]. It has been hypothesized that Fhb1 of ‘Sumai3’ is in fact a UDP-glucosyltransferase [145]. Fine-mapping of Fhb1 by James Anderson’s research group in Minnesota have narrowed it down to a few candidate genes. Surprisingly, none of these encode UDP-glucosyltransferase, but this does not eliminate the possibility that Fhb1 is involved in the regulation of UDP-glucosyltransferase expression [112]. Differential accumulation of UDP-glucosyltransferase transcript and protein has been observed in wheat (Foroud *et al*., in preparation) and maize (Harris *et al*., in preparation), respectively. Transgenic expression of UDP-glucosyltransferase might lead to improved FHB-resistance and reduced toxin accumulation; however, this approach may be dangerously misleading. Studies have shown than glycosylated-ZON can be reconverted to ZON in the intestinal tract of swine [146].

## 4. Conclusions

Trichothecene contamination of cereals represents a threat to the economies and food supplies of cereal growing regions around the world. More importantly, in countries where there is limited or no screening for trichothecene contamination, it represents a significant health threat. We would like to make three recommendations to breeders and scientists to help reduce the threat of trichothecene-induced damages. The first of these is already a goal most breeders strive towards, and that is to breed for highly-resistant cultivars with good agronomic traits. Highly-resistant cultivars express stable resistance under epidemic conditions, moderate FHB-resistance is not good enough. Highly-resistant cultivars are of little use if farmers choose not to grown them, and so good agronomics in combination with resistance is essential.

Our second recommendation is one recently made by Boutigny *et al*., and that is to breed for type V resistance [83]. While trichothecene screening is costly and time consuming in breeding programs, resistance to DON-accumulation is of utmost importance. Most other forms of resistance may be improved in the presence of type V resistance, and type V resistance may be a consequence of other forms of resistance; however, all other forms of resistance are irrelevant if trichothecenes accumulate in the grain. Moreover, breeding for type V-2 (by inhibition of trichothecene biosynthesis) is preferred over type V-1 (chemical modification of trichothecenes), since it is unclear whether the modified trichothecenes remain detoxified upon ingestion. While distinguishing between the two classes of type V resistance may present more time and cost to breeders, these inconveniences can be reduced following recommendations by Miedaner *et al*. [31]. That is to screen first for FHB-severity, and once the breeding lines have been narrowed down screen for resistance to trichothecene accumulation. DON quantification by ELISA is much more affordable than GC-MS or LC-MS/MS, but the advantage of mass spectrophotometry-based techniques is that multiple trichothecenes can be detected, and glycosylated derivatives can also be detected [147–153]. *In vitro* selection is a very effective method to select for good resistance to trichothecenes, though sources of resistance have to be adequately identified. As well, these early screening methods should be used in combination with disease rating in controlled environments and could be applied with molecular marker assisted selection for the major QTLs.

Finally, our third recommendation to reduce the impact of trichothecene contamination in the food-supply is to breed for resistance in “other crops”—that is to say in addition to wheat and barley. Most breeding efforts/successes have been reported in wheat and barley, especially wheat, but there are many other crops (such as maize, oats, rye and triticale) which are affected by FHB and accumulate trichothecenes that do not get as much attention as they may deserve. Breeding for resistance to FEB in maize is of particular importance, since maize crop residue is known to be a major source of soil-borne inoculum. The presence of type A trichothecenes (which are not usually screened for in food products) in oat and oat products as a result of infection with *F. langsethiae* [104, 173] emphasizes the need to breed more resistant oats—even though this crop is not typically seen as highly susceptible in comparison to wheat, barley and corn. Disease in these crops leads not only to contamination in the food chain, but also to inoculum build-up in the soil, which subsequently leads to increased epidemics and increased risk of trichothecene contamination within our food supply. The development and the farming of highly resistant cultivars (resistant to the disease and to trichothecene accumulation), in all cereal crops affected by this disease, will go a long way in protecting the consumer from health hazards associated with ingestion of trichothecene-contaminated grain, and in protecting farmers from the economic impacts of this disease.

## Figures and Tables

**Table 1.** Genes involved in trichothecene production. For more details on trichothecene gene cluster see [163, 167–170]. Note that recent analyses have revealed that *Tri1* and *Tri101* are in the core-trichothecene gene cluster (core-*Tri*) in *F. equiseti* and *F. scirpi* (R. Proctor, unpublished).

Gene	Cluster	Description	References
***Enzymes****: for pathway reactions see also Scheme 1*			
*Tri1*	*Tri1-Tri16*	C-7 monooxygenase (*F. graminearum*); C-8 monooxygenase (*F. graminearum, F. sporotrichioides*)	[154–156, 174–175]
*Tri3*	Core *Tri*	15-*O*-acetyltransferase	[47]
*Tri4*	Core *Tri*	monooxygenase	[41–42, 157]
*Tri5*	Core *Tri*	sesquiterpene cyclase, ‘trichodiene synthase’ 4-*O*-acetyltransferase; functional *F. graminearum* TRI7 required for	[6, 40, 158]
*Tri7*	Core *Tri*	NIV-chemotype; functional *F. sporotrichioides* TRI7 required for T-2 toxin production	[49–50]
*Tri8*	Core *Tri*	C-3 deacetylase; functional *F. sporotrichioides* TRI8 required for T-2 toxin production	[50, 159]
*Tri9*	Core *Tri*		[50]
*Tri11*	Core *Tri*	C-15 monooxygenase	[45, 160]
*Tri13*	Core *Tri*	monooxygenase; functional *F. graminearum* TRI13 required for NIV-chemotype	[49, 161]
*Tri14*	Core *Tri*		
*Tri16*	*Tri1-Tri16*		[162]
*Tri101*	None	15-*O*-acetyltransferase	[44, 136, 162]
***Transcription Factors***			
*Tri6*	Core *Tri*	zinc-finger DNA binding protein; required for T-2 toxin production; binding motif (YNAGGCC) found in most promoter regions within Tri5 cluster	[164, 165, 50]
*Tri10*	Core *Tri*		[166]
***Other***			
*Tri12*	Core *Tri*	major facilitator superfamily (MFS) transporter involved in trichothecene efflux	[166, 141]

**Table 2.** FHB resistance mechanisms in cereals.

Resistance	Description
***Resistance in Small Grain Cereals (as defined in reference 99)***	
Type I	Resistance to initial infection [90]
Type II	Resistance to disease spread [90]
Type III	Resistance to kernel infection [87]
Type IV	Tolerance against FHB and trichothecenes [87]
Type V	Resistance to trichothecene accumulation [97]
class 1	by chemical modification of trichothecenes [83]
class 2	by inhibition of trichothecene synthesis [83]
***Resistance in Maize***			
Silk Resistance	Resistance to silk penetration [88]
Kernel Resistance	Resistance to kernel disease spread [89]
